# Supplementary material for: KCML: a machine‐learning framework for inference of multi‐scale gene functions from genetic perturbation screens
Source: Mol Syst Biol. 2020 Mar 6;16(3):e9083. doi: 10.15252/msb.20199083 (PMC7059140; doi:10.15252/msb.20199083)
Supplement: Supplementary file 1 — Expanded View Figures PDF [file MSB-16-e9083-s001.pdf]

Expanded View Figures

Figure EV1. Single-cell image-based data allows dissecting perturbation effects that are independent of cell number and complements other types of phenotypic data.

A Estimated density of the number of cells across 18,033 gene knockdowns.

B, C Scatter plots of TCN (total cell number) versus Nuclei area mean (B) and II mitotic (infection index of mitotic cells) before and after correction for dependency on TCN (C). R^2 indicates Pearson correlation coefficient. Colour indicates number of cells (0–2,000, . . . 18,000–20,000).

D Box plots of the correlation between SVM confidence scores and cell number before and after TCN correction. Box plots elements: centre line, median; box limits, 25th and 75th percentiles; whiskers, $\pm$ 2.7 standard deviation. Points: outliers ($n = 145$).

E Individual AUROC curves for the different GO term classifiers for the three datasets.

F GO terms where single-cell-resolved image-based readouts outperform expression readouts based on overall recall.

G GO terms that are only classifiable based on the image-based dataset.

H Precision–recall curves for the three datasets where the shaded area indicates the 95% confidence interval (left). The same is shown on the right but when considering first neighbours of annotated genes for a given GO term in the protein–protein interaction network as true-positives.

 © 2020 The Authors

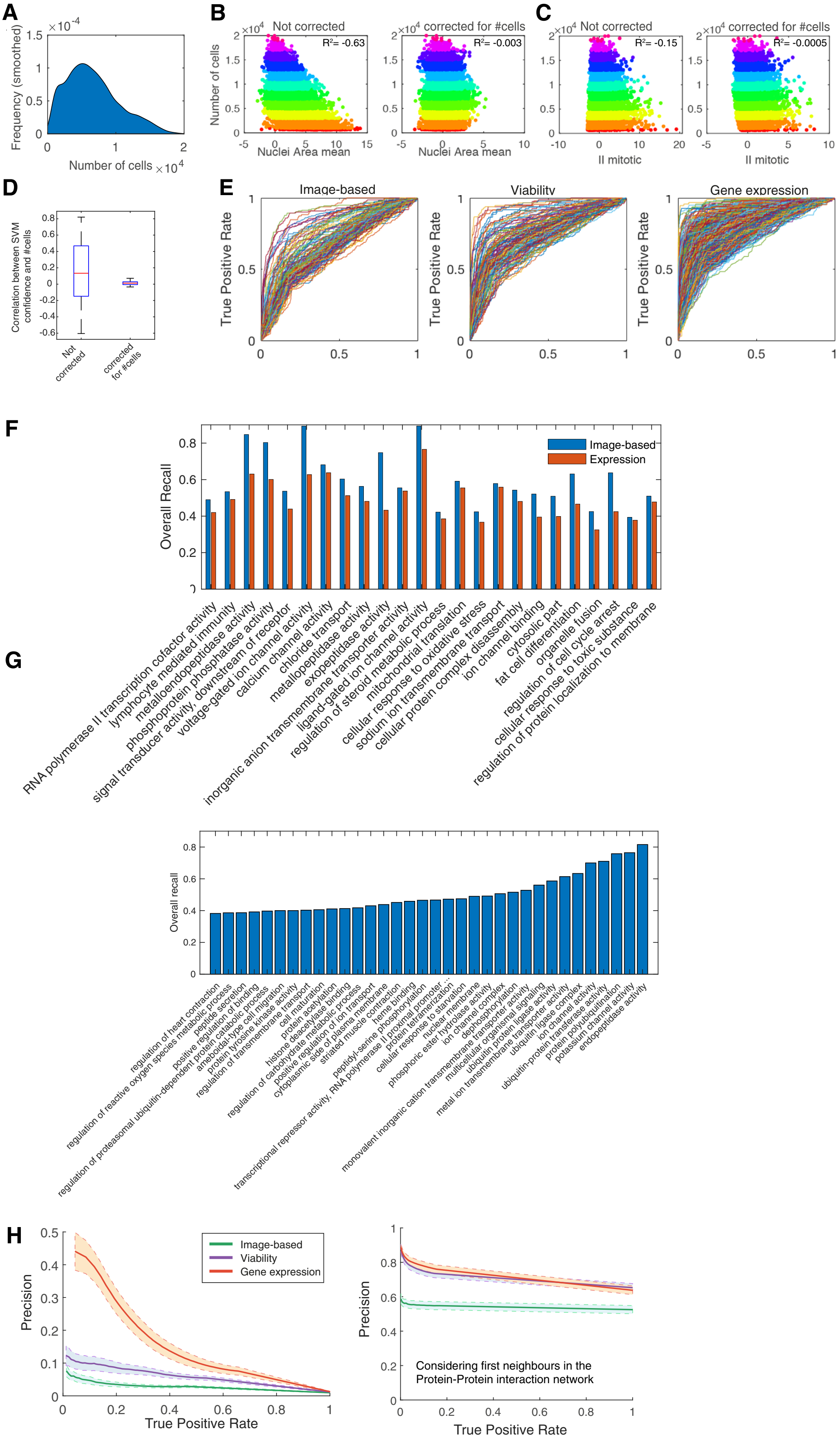

Figure EV1.

Figure EV2. The predictions of GO term classifiers have a moderate overlap and select a diverse range of features.

A The recall on test data for terms that are classifiable based on both infection and shape features.

B, C Clustering (B) and distribution (C) of Jaccard index values based on the overlap between predictions of GO term classifiers. Most terms have a moderate overlap suggesting that different phenotypes are discovered.

D The number of features in different categories that are selected by the respective GO term classifier (scaled) when the classifier is trained using (i) only shape features or (ii) only infection features. Blue indicates the number of features with a higher average than control, while red indicates the number of features with a lower average than control.

E Feature categories based on feature type (e.g., morphology, cell context, DAPI intensity), measurement type (e.g., summary, spread, distribution shape). The topmost frequently selected features by different GO term classifiers are shown as examples if applicable. Hue of orange circles indicates feature importance based on the number of classifiable terms where the corresponding feature was selected.

© 2020 The Authors

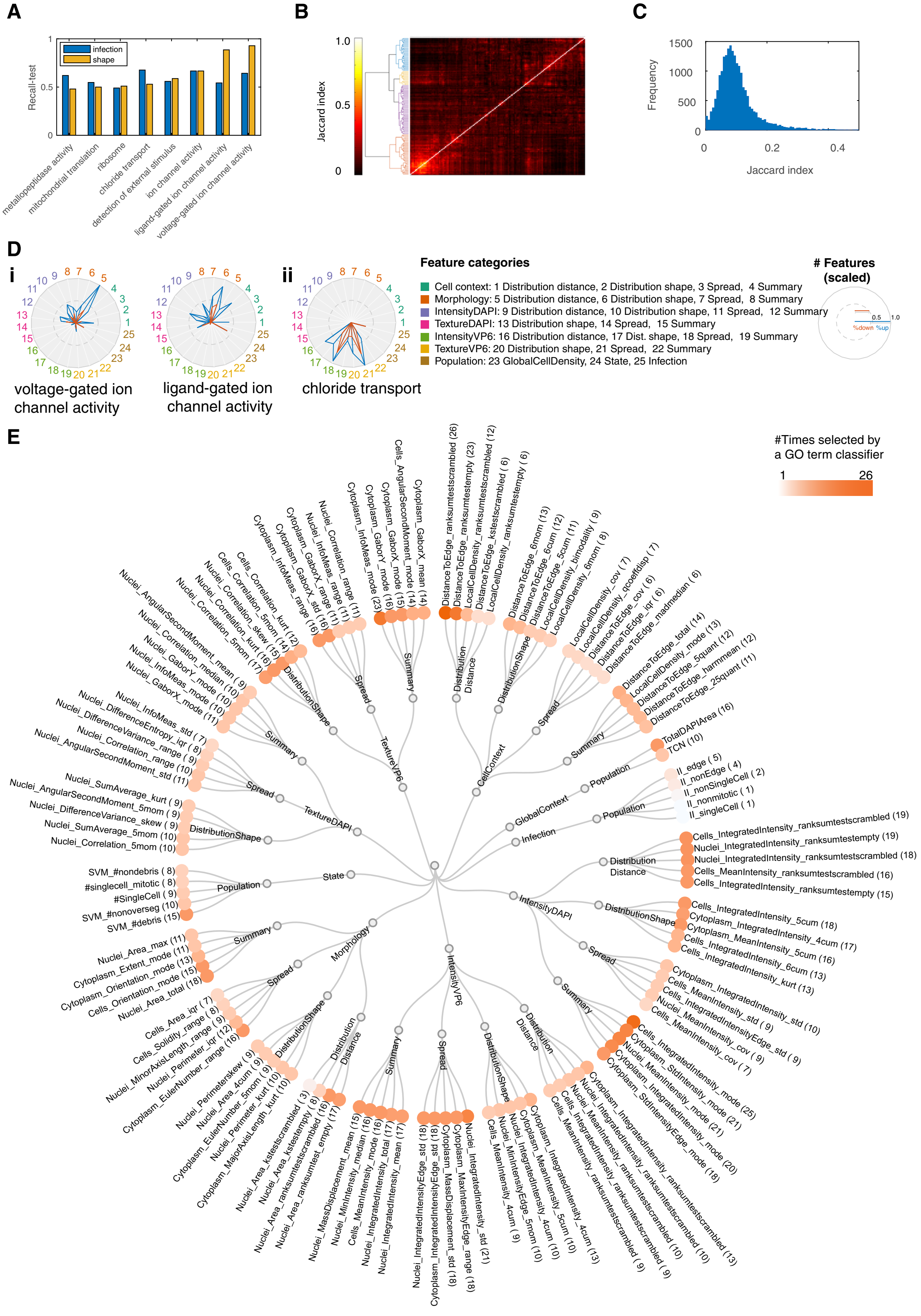
A
infection
shape
Recall-test
1
0.5
0
metallopeptidase activity
mitochondrial translation
ribosome
chloride transport
detection of external stimulus
ion channel activity
ligand-gated ion channel activity
voltage-gated ion channel activity
B
1.0
0.5
0
Jaccard index
C
1500
1000
500
0
Frequency
0
0.2
0.4
Jaccard index
D
i
ii
voltage-gated ion channel activity
ligand-gated ion channel activity
chloride transport
Feature categories
Cell context: 1 Distribution distance, 2 Distribution shape, 3 Spread, 4 Summary
Morphology: 5 Distribution distance, 6 Distribution shape, 7 Spread, 8 Summary
IntensityDAPI: 9 Distribution distance, 10 Distribution shape, 11 Spread, 12 Summary
TextureDAPI: 13 Distribution shape, 14 Spread, 15 Summary
IntensityVP6: 16 Distribution distance, 17 Dist. shape, 18 Spread, 19 Summary
TextureVP6: 20 Distribution shape, 21 Spread, 22 Summary
Population: 23 GlobalCellDensity, 24 State, 25 Infection
Features (scaled)
0.5
1.0
%down
%up
E
#Times selected by a GO term classifier
1
26
CellContext
GlobalContext
Infection
IntensityDAPI
IntensityVP6
Morphology
State
TextureDAPI
TextureVP6
Population
Summary
Spread
DistributionShape
Distribution Distance

Figure EV2.

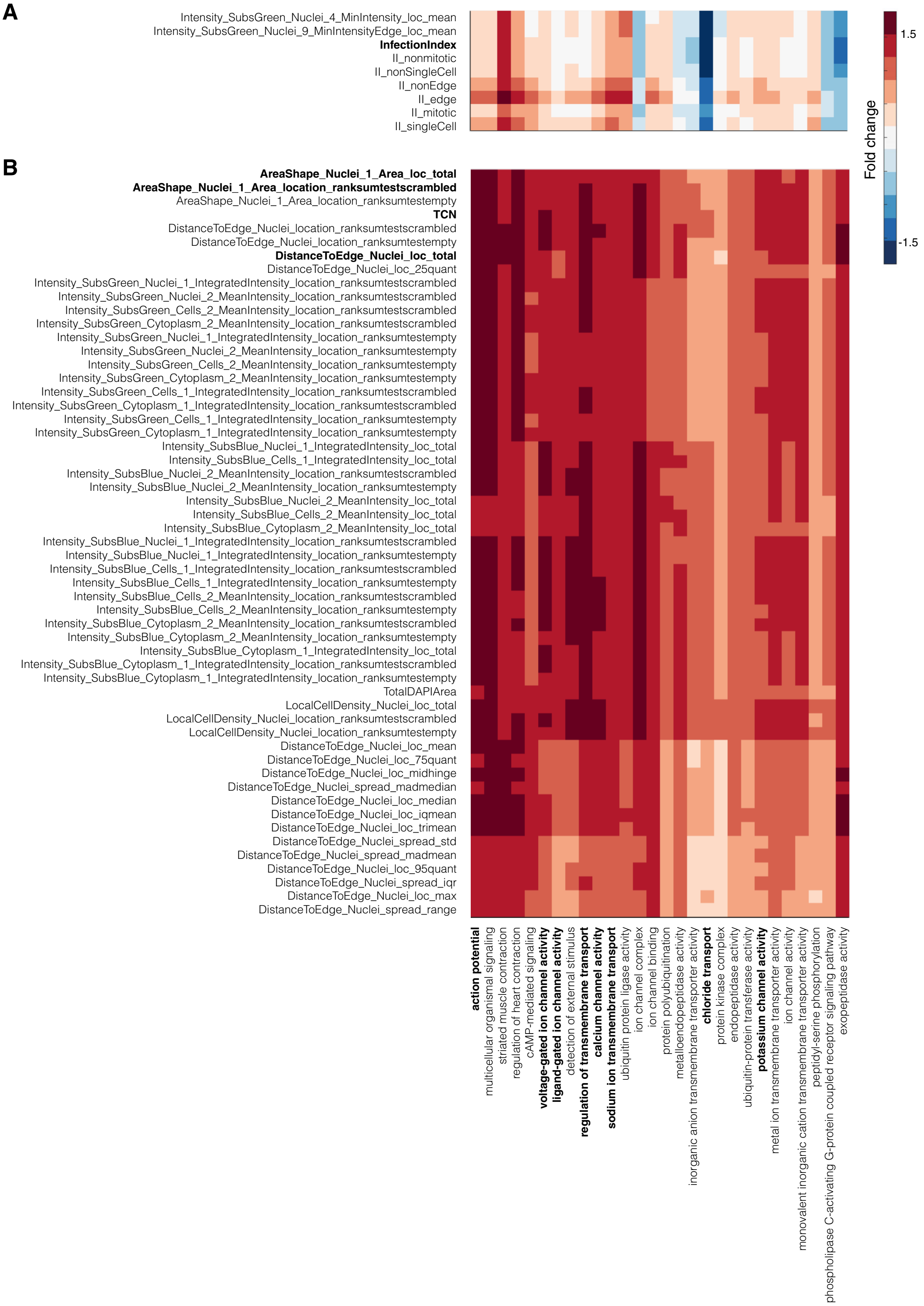

Figure EV3. Morphology and microenvironment features are predictive of many GO terms involved in membrane transport.

A, B Examples of significantly changed features for terms in Fig 4A (C1) which include many membrane transport terms. TCN: total cell number.

 © 2020 The Authors

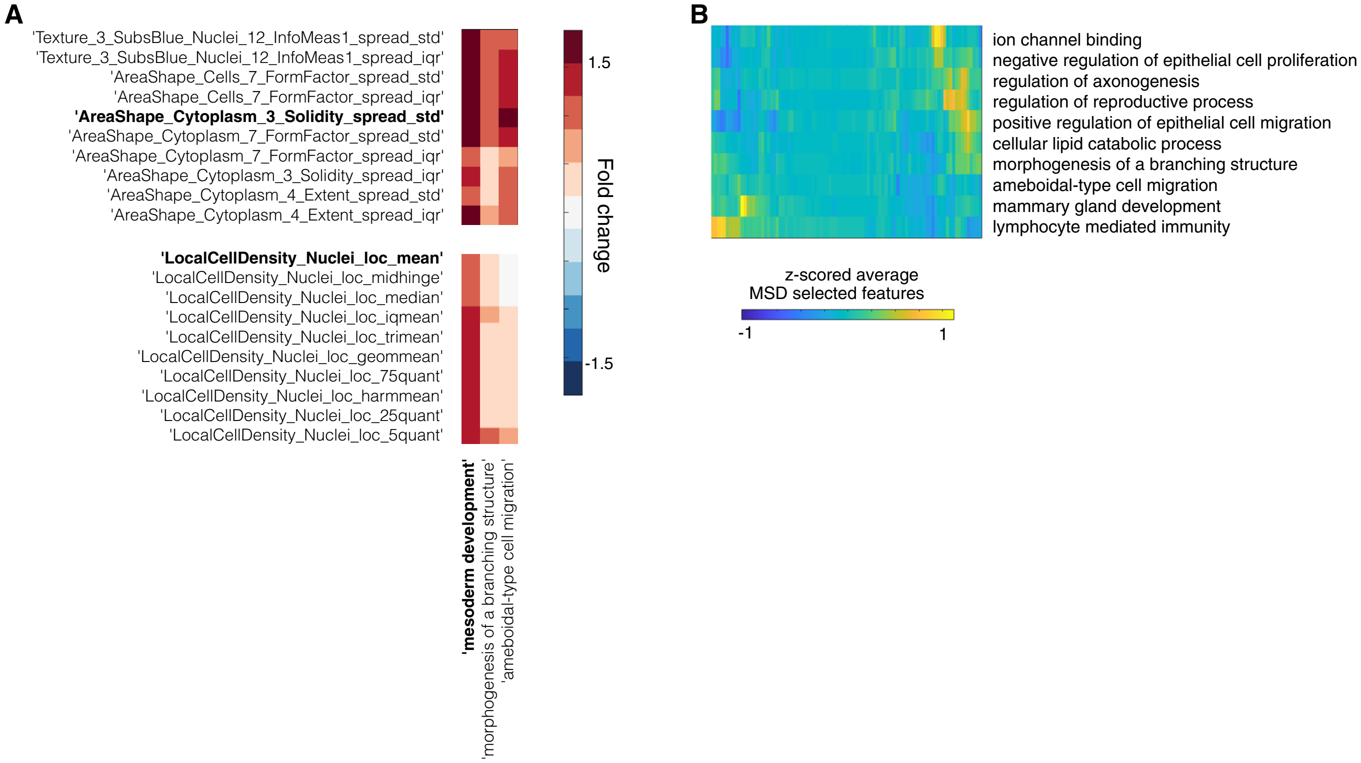

Figure EV4. Microenvironment and heterogeneity measurements are predictive of Mesoderm Development GO term and their variation can specify lower-scale functions.

A Example of significantly changed features for terms in Fig 4A (C10) that include MSD.
B Average values of features selected by MSD classifier for genes predicted to perform additional functions other than MSD.

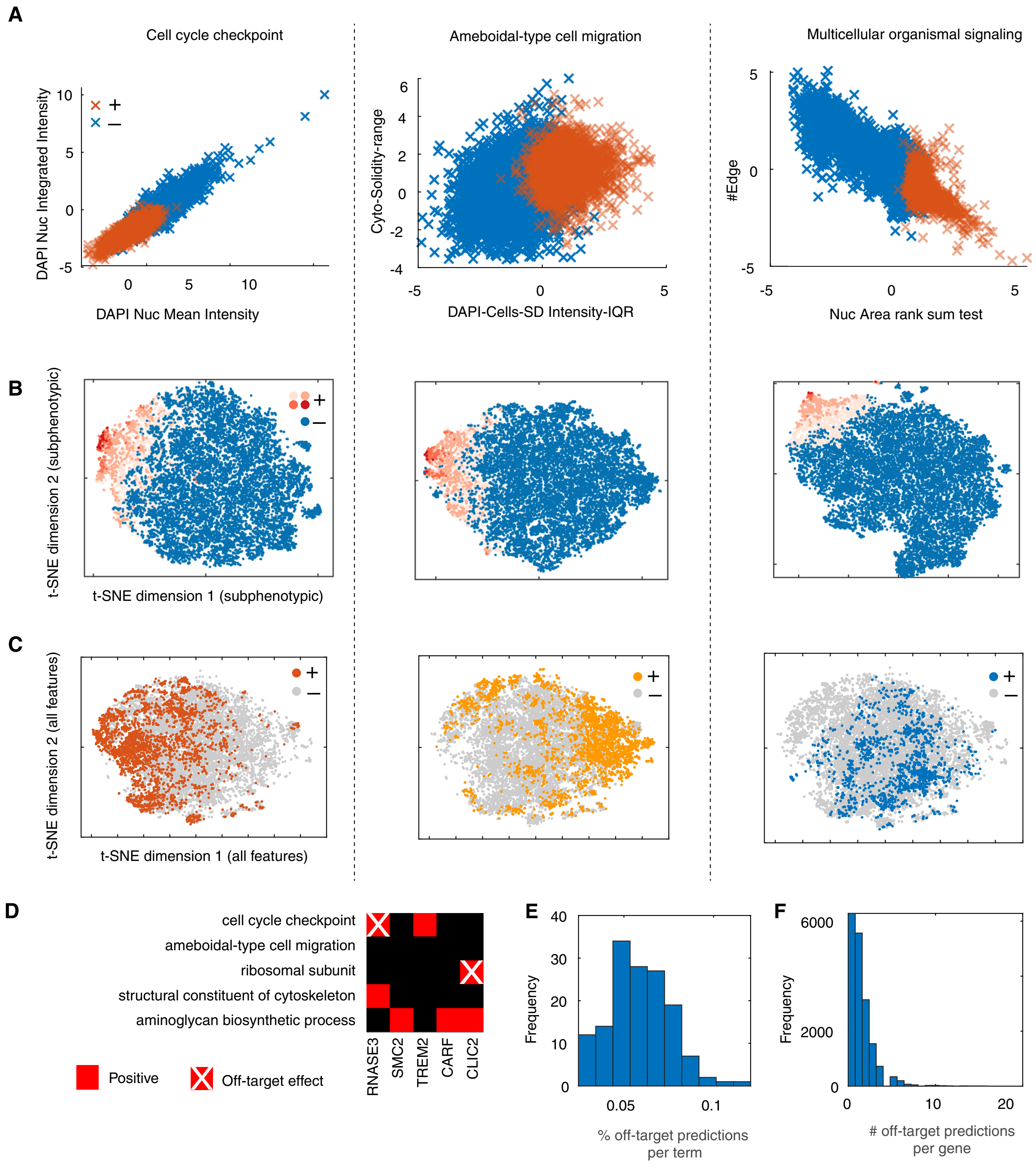

Figure EV5. Deconvolution of sub-phenotypic sapces and off-target effects.

A–C Comparison between negative and positive genes predicted by the respective GO term classifier based on: two significantly changed features (A), subphenotypic t-SNE embedding (based on the selected features by the respective classifier) where red hues indicate SVM rank (B), or phenotypic t-SNE embedding (based on all measured features) (C).

D Example of predicted functions for five genes and the detected off-target effects based on siRNA seed enrichment.

E, F Distribution of the number of off-target predictions per GO term classifier (E) or per gene (F).

© 2020 The Authors

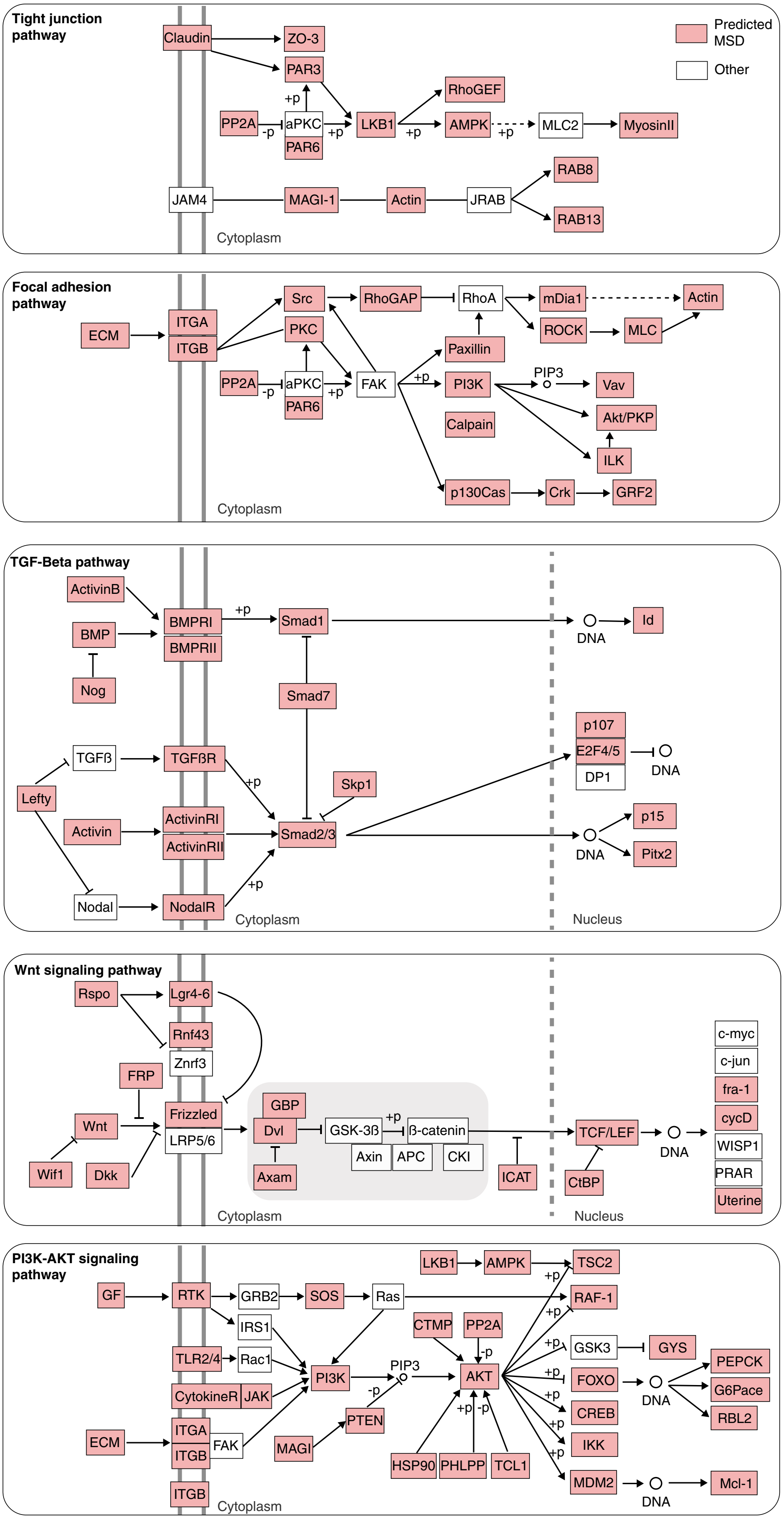

Figure EV6. Predictions of MSD classifier are significantly enriched for cell adhesion and colorectal cancer pathways.

Snapshots from tight junctions, focal adhesion, TGFβ, WNT and PI3K-AKT KEGG pathways where MSD genes are highly represented. Predicted MSD genes are indicated in red nodes.

© 2020 The Authors

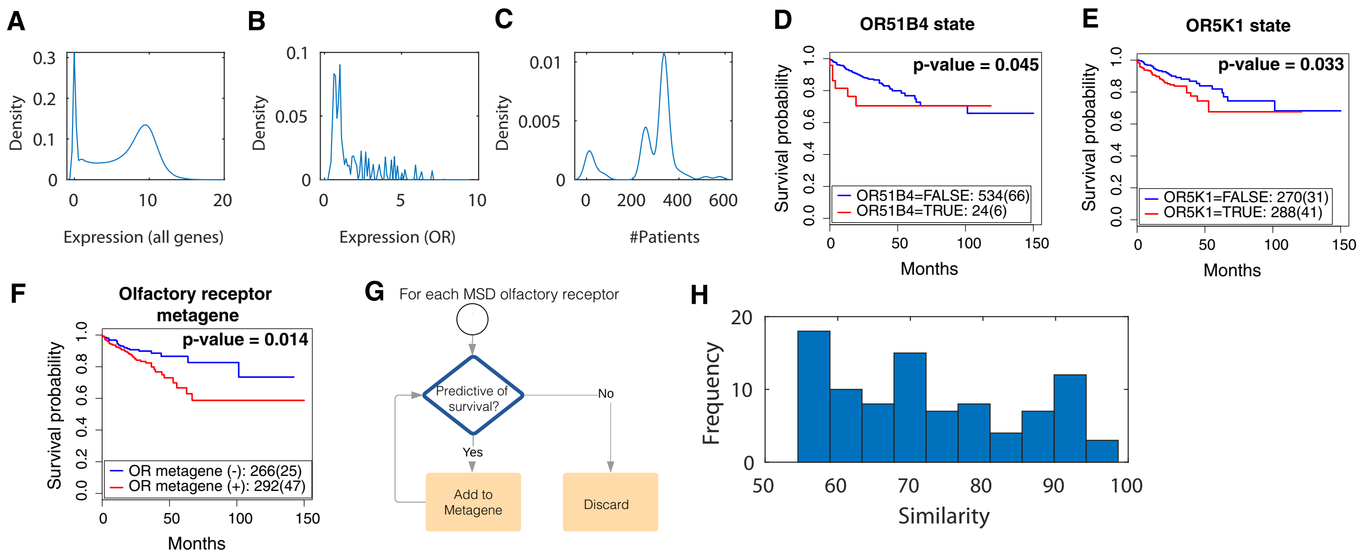

Figure EV7. Most olfactory receptors have low expression in colorectal cancer patients but correlate with patient survival.

A, B Distributions of the expression of all genes (A) and MSD-associated olfactory receptors (B) in colorectal cancer patients based on TCGA.

C Distribution of the number of patients where an MSD-associated olfactory receptor is expressed.

D–F Kaplan–Meier survival analysis of colorectal cancer patients based on the expression state of *OR51B4* (D), *OR5K1* (E) and MSD-associated olfactory receptor metagene (F). OR: olfactory receptor.

G Derivation of MSD-associated olfactory receptor metagene where the expression state of each receptor is added iteratively to the metagene and retained if scored significant based on Kaplan–Meier survival test (validated by leave-one-patient-out).

H Sequence similarity between olfactory receptors in our metagene with other olfactory receptors based on the human olfactory data explorer (Olender *et al*, 2013).

 © 2020 The Authors
