# Supplementary material for: KCML: a machine‐learning framework for inference of multi‐scale gene functions from genetic perturbation screens
Source: Mol Syst Biol. 2020 Mar 6;16(3):e9083. doi: 10.15252/msb.20199083 (PMC7059140; doi:10.15252/msb.20199083)
Supplement: Supplementary file 11 — Code EV1 [file MSB-16-e9083-s011.zip › SupplementarySoftware/Source/readme.rtf]

KCML predicts gene functions using perturbation phenotypic data. KCML optimise and train an SVM classifier for each gene ontology term but it can be applied to any type of annotations the user define. Requirements:- desired values in config.txt including experiment name, sigma values, and number of cross validation during training.- annotations files in annotations folder. The first column should have gene names and the second column should have functional annotation name as in the included example file. The name of this file should follow the following format: $expName$Annotation. For example if expName=Demo then annotation file name should be DemoAnnotation.csv.- data files in annotations folder. The first column should have gene names as in the included example file.The name of this file should follow the following format: $expName$Data. For example if expName=Demo then data file name should be DemoData.csv.Output:- The prediction of KCML on various functional terms and its confidence in these predictions - The performance of the classifiers- The selected features by different classifiersIf you use this program, please citeSalem H., Rittscher J., Pelkmans L., 2019, KCML: a machine-learning framework for inference of multi-scale gene functions from genetic perturbation screens, bioRxiv 761106
